# Targeting Mitochondrial Oxidative Phosphorylation Abrogated Irinotecan Resistance in NSCLC

**DOI:** 10.1038/s41598-018-33667-6

**Published:** 2018-10-24

**Authors:** Soohyun Lee, Jae-Seon Lee, Jinho Seo, Seon-Hyeong Lee, Joon Hee Kang, Jaewhan Song, Soo-Youl Kim

**Affiliations:** 1 Tumor Microenvironment Research Branch, Division of Cancer Biology, Research Institute, National Cancer Center, Goyang, 10408 Republic of Korea; 2 Department of Biochemistry, College of Life Science and Biotechnology, Yonsei University, Seoul, 03722 Korea

## Abstract

Anticancer drug resistance is a major challenge of cancer therapy. We found that irinotecan-resistant NSCLC cells showed increased mitochondrial oxidative phosphorylation compared to the drug sensitive NSCLC cells. Previously, we found that combined inhibition of aldehyde dehydrogenase using gossypol, and mitochondrial complex I using phenformin, effectively reduced oxidative phosphorylation in NSCLC. Here, we showed that targeting oxidative phosphorylation with gossypol and phenformin abrogated irinotecan resistance in NSCLC. Furthermore, irinotecan treatment by blocking oxidative phosphorylation induced synergistic anti-cancer effect in NSCLC. The pre-clinical xenograft model of human NSCLC also demonstrated a therapeutic response to the dual targeting treatment. Therefore, this combination of gossypol and phenformin increases irinotecan sensitivity as well as preventing irinotecan resistance.

## Introduction

Anti-cancer drug resistance may be acquired from clonal selections of resistant variants with adaptive tumor responses. Although targeting cancer specific markers prolongs survival of cancer patients, it is still far from a non-recurrent cure in NSCLC^1^. Recurrent cancer often showed drug resistance following anti-cancer drug treatment. Some theories have been suggested, including clonal evolution^2,3^, but an alternative therapeutic strategy is not available. Chemotherapy-resistant human acute myeloid leukemia showed increased mitochondrial mass and retained active mitochondria with a high level of oxidative phosphorylation (OxPhos)^4,5^. In drug-resistant solid cancer, MYC and MCL1 are frequently co-amplified after neoadjuvant chemotherapy, which are responsible for drug resistance through increase of mitochondrial OxPhos^6^.

Recently, we have shown that ATP production through OxPhos in NSCLC can be stalled down to 20% of the control, by combination treatment of inhibitions for aldehyde dehydrogenase (ALDH) and mitochondrial electron transfer complex I using gossypol and phenformin^7–9^. The suggested mechanism of ATP depletion was based on the cancer cell using cytosolic NADH produced by ALDH as an electron source for ATP synthesis through OxPhos^7,8,10^. Normal cells are not affected by gossypol and phenformin, because the cells use mitochondrial NADH produced from the TCA cycle. Therefore, gossypol and phenformin did not affect ATP production in normal cells, or the growth of normal body weight during treatment^7,10^. Here we observed that irinotecan resistant NSCLC cells showed increased mitochondrial OxPhos. Therefore, we have tested whether blocking OxPhos using gossypol and phenformin may reverse drug resistance in NSCLC.

## Materials and Methods

### Cell lines

H1975 (ATCC CCL-5908, Manassas, VA, U.S.A.), H23 (ATCC CCL-5800, Manassas, VA, U.S.A.), H226 (ATCC CCL-5826, Manassas, VA, U.S.A.) and IMR-90 (normal lung fibroblast, ATCC CCL-186, Manassas, VA, U.S.A.) were purchased from ATCC. Other NSCLC cell lines were obtained from the U.S. National Cancer Institute (Bethesda, MD, U.S.A.) (MTA 1-2702-09).

### Cell culture

All NSCLC cells were grown in RPMI 1640 medium (SH30027.01, HyClone, Logan, UT, U.S.A.) containing 10% fetal bovine serum (FBS) (SH30070.03HI, HyClone, Logan, UT, U.S.A.), penicillin, and streptomycin. IMR-90 cell was grown in DMEM/HIGH GLUCOSE medium (SH30243.01, Hyclone, Logan, UT, U.S.A.) containing 10% FBS. Cells were incubated at 37 °C and maintained at 5% CO_2_. siRNA duplexes targeting human ALDH1L1 were transfected into cells for 72 h using Lipofector-Q Reagent (AB-LF-Q001, AptaBio, Yongin, Korea) and Plusfector Reagent (AB-PF-0001, AptaBio, Yongin, Korea) according to the manufacturer’s instructions. As negative controls, cells were incubated with Lipofector-Q Reagent, Plusfector Reagent and a negative siRNA (sc-37007, sc-44230) (Santa Cruz, Dallas, TX, U.S.A.). The ALDH1L1 siRNA sequences are in Table 1 below:

**Table 1:** The ALDH1L1 siRNA sequences

No	Gene Name	sequence	
		sense	antisense
1	ALDH1L1 #1	GAGUGCCGGUAUUCAAGUAdTdT	UACUUGAAUACCGGCACUCdTdT
2	ALDH1L1 #2	CCAUAAGUAACGUGAAGAAdTdT	UUCUUCACGUUACUUAUGGdTdT

### Sulforhodamine B assay: cell growth assay

Cells (100 μL) were inoculated into 96-well microplates at plating densities ranging from 5,000 to 20,000 cells/well depending on their doubling time. After cell inoculation, the microplates were incubated for 24 h prior to the addition of the experimental drugs or siRNA transfection. The drugs were prepared and 100 μL was added to each well so that the final concentrations were as indicated in the figures; the plates were then incubated in CO_2_ incubator. At the same time, control plates (T0) were fixed by adding 50 μL 10% (w/v) TCA and incubated at 4 °C. After 48 h of treatment, cells were fixed by adding TCA and incubated for 60 min at 4 °C. The plates including T0 were washed five times with tap water and air dried. Sulforhodamine B (SRB) solution (100 μL) at 0.4% (w/v) in 1% acetic acid was added to each well, and the plates were kept for 5 min at room temperature. After staining, the plates were washed five times with 1% acetic acid to remove the unbound dye and air dried. The bound stain was solubilized with subtracting the obtained values from the values of T0 plates and the percentage was calculated.

### Western blot analysis

Harvested cells were lysed with RIPA cell lysis buffer in the presence of protease and phosphatase inhibitor cocktail (Sigma, St. Louis, MO, U.S.A.). The protein concentration of the cell lysates was quantified by a BCA Pierce Protein Assay Kit (Thermo Fisher Scientific, Waltham, MA, U.S.A.). The same amount of protein samples was loaded onto 10% SDS-PAGE and transferred onto PVDF membranes. After blocking by 5% BSA, the membranes were incubated in the primary antibodies diluted in 5% BSA buffer for overnight at 4 °C and then in the HRP-conjugated secondary antibody for 1 h at room temperature. The protein band images were captured with ECL reagent (Thermo Fisher Scientific, Waltham, MA, U.S.A.). The primary antibodies used in the experiments were ALDH1L1 (UM500039, OriGene, Rockville, MD, U.S.A.), β-actin (sc-47778, Santa Cruz, Dallas, TX, U.S.A.) and OxPhos cocktail (ab110411, Abcam, Cambridge, U.K.).

### XF Cell Mito Stress Analysis

To test the effect of irinotecan on cellular respiration of A549 irinotecan-resistant cells, A549 and A549R cells were plated in each well of a seahorse microplate and after 24 h, cells were treated with 5 μM gossypol and 100 μM phenformin for 24 h. For oxygen consumption rate (OCR) determination, cells were incubated in XF base medium supplemented with 10 mM glucose, 1 mM sodium pyruvate and 2 mM L-glutamine and equilibrated in non-CO_2_ incubator at 37 °C for 1 h before starting the assay. The samples were mixed (3 min) and measured (3 min) using XFe96 extracellular flux analyzer (Seahorse Bioscience, Billerica, MA, U.S.A.). Oligomycin (1 μM), FCCP (1 μM), and rotenone/antimycin A (0.5 μM) dissolved in XF base medium were injected at the indicated time points. Oligomycin, FCCP, rotenone, and antimycin A were purchased from Sigma-Aldrich. Raw data were normalized to protein concentration by the SRB assay.

### Measurement of mitochondrial membrane potential (∆ψm) (TMRE)

Cells were cultured into 100 mm dishes for flow cytometry or into coverglass bottom 6-well plates for live cell imaging. After 24 h, cells were treated with drugs as indicated. Twenty minutes prior to the end of each treatment, 100 nM tetramethylrhodamine-ethylester (TMRE; ab113852; Abcam, Cambridge, U.K.) was added to the culture medium. Cells were washed three times with ice-cold PBS. Images were captured with Axio Observer Z1 microscope (Carl Zeiss, Oberkochen, Baden-Württemberg, Germany) or analyzed by FACSCalibur flow cytometry (BD Falcon, Bedford, MA, USA).

### Annexin V-FITC apoptosis detection

Cells were treated as indicated for 48 h, washed twice in cold PBS, centrifuged at 1,400 rpm for 3 min, and Annexin V and propidium iodide (PI) double staining was done using Annexin V-FITC apoptosis detection kit (ALX-850-020-KI01, Enzo, Farmingdale, NY, U.S.A.) according to the manufacturer’s instructions. The samples were analyzed by FACSCalibur flow cytometry (BD Falcon, Bedford, MA, U.S.A.).

### TUNEL assay: Cell death detection

A fluorometric TUNEL detection kit was used according to the manufacturer’s instructions (11684795910; Roche Applied Science, Indianapolis, IN, U.S.A.). In brief, cells were treated as indicated and fixed with 4% paraformaldehyde in PBS for 10 min, permeabilized with 0.5% Triton X-100 in PBS for 2 min at 4 °C and incubated with the provided fluorescein-conjugated TUNEL reaction mixture in a humidified chamber at 37 °C for 1 h in dark. Omission of the addition TdT enzyme in the TUNEL reaction mixture was included as negative control. The cells were then mounted with 4′,6-diamidino-2-phenylindole (DAPI) mounting medium to visualize nuclei (Vectashield mounting medium; Vector Laboratories, Burlingame, CA, U.S.A.). TUNEL- and DAPI-stained nuclei staining were examined under a Zeiss LSM780 confocal microscope (Carl Zeiss, Oberkochen, Baden-Württemberg, Germany).

### Cell Titer-Glo Luminescent Cell Viability Assay: Cell death analysis

A549 cells were incubated with combination of irinotecan, gossypol, phenformin, z-VAD-fmk, necrostatin-1, ferrostatin-1 and 3-MA for indicated times. After incubation, treated cells were imaged by microscope and analyzed by Cell Titer-Glo Luminescent Cell Viability Assay (Promega) according to the manufacturer’s instructions. Cell Loss was determined by following calculation: cell loss percentage = (1 − ATP_sample_/ATP_control_) × 100.

### Measurement of Mitochondrial Membrane Potential (Mitotracker Green FM)

Cells were seeded on coverslips and after 24 h, media containing 200 nM Mitotracker FM green probe (M7514, Thermo Fisher Scientific) was added for 45 min. Cells were fixed with 4% (w/v) paraformaldehyde for 10 min and permeabilized with 0.5% Triton X-100 for 10 min. The cells were then mounted with 4′,6-diamidino-2-phenylindole (DAPI) mounting medium to visualize nuclei (Vectashield mounting medium; Vector Laboratories, Burlingame, CA). Samples were examined under a Zeiss LSM780 confocal microscope (Carl Zeiss, Oberkochen, Baden-Württemberg, Germany).

### Preclinical xenograft tumor models

Balb/c-nu mice (Orient, Seoul, Korea) were aged between 6 and 8 weeks before tumor induction. This study was reviewed and approved by the Institutional Animal Care and Use Committee (IACUC) of the National Cancer Center Research Institute, which is an Association for Assessment and Accreditation of Laboratory Animal Care International (AAALAC International) accredited facility that abides by the Institute of Laboratory Animal Resources guide (protocols: NCC-18-425). All methods were performed in accordance with the relevant guidelines and regulations. A549 cells (7.5 × 10^6^) in 100 μL PBS were subcutaneously inoculated using a 1-mL syringe. After a week, the mice were divided into groups as indicated. Vehicle (5% DMSO and 5% cremophor in PBS, 100 μL) alone, gossypol (80 mg/kg/100 μL), and phenformin (100 mg/kg/100 μL) were administered orally once per day, 6 days/week, and irinotecan (20 mg/kg/100 μL) or gemcitabine (80 mg/kg/100 μL) was administered i.p. once per day, 1 day/week (n = 8). Primary tumor size was measured every week using calipers. Tumor volume was calculated using the formula, V = (A × B^2^)/2, where V is the volume (mm^3^), A is the long diameter, and B is the short diameter.

The experiment was repeated with H1975 cells (5 × 10^6^) with higher concentration of irinotecan (40 mg/kg/100 μL) and were divided into four groups when the size of tumors reached approximately 1000 mm^3^ (n = 5).

### Immunohistochemistry

Formaldehyde (4%) fixed specimens were paraffin-embedded and cut at a thickness of 4 μm. Sections were dried for 1 h at 56 °C and immunohistochemical staining performed with the automated instrument Discovery XT (Ventana medical system, Tucson Arizona, USA) using the Chromomap DAB Detection kit as follow: sections were deparaffinized and rehydrated with EZ prep (Ventana) and washed with Reaction buffer (Venatana). The antigens were retrieved with heat treatment in pH 6.0 Citrate buffer (Ribo CC, ventana) at 90 °C for 30 min for anti-Ki-67 (ab15580, Abcam, Cambridge, U.K.).

### Statistical Analysis

Statistical analysis was performed using the Student’s t test as appropriate. Tumor growth was analyzed statistically by two-way analysis of variance (ANOVA) tests using GraphPad PRISM 5.

## Results

### Mitochondrial OxPhos was highly increased in irinotecan resistant NSCLC cells

**Figure 1:**
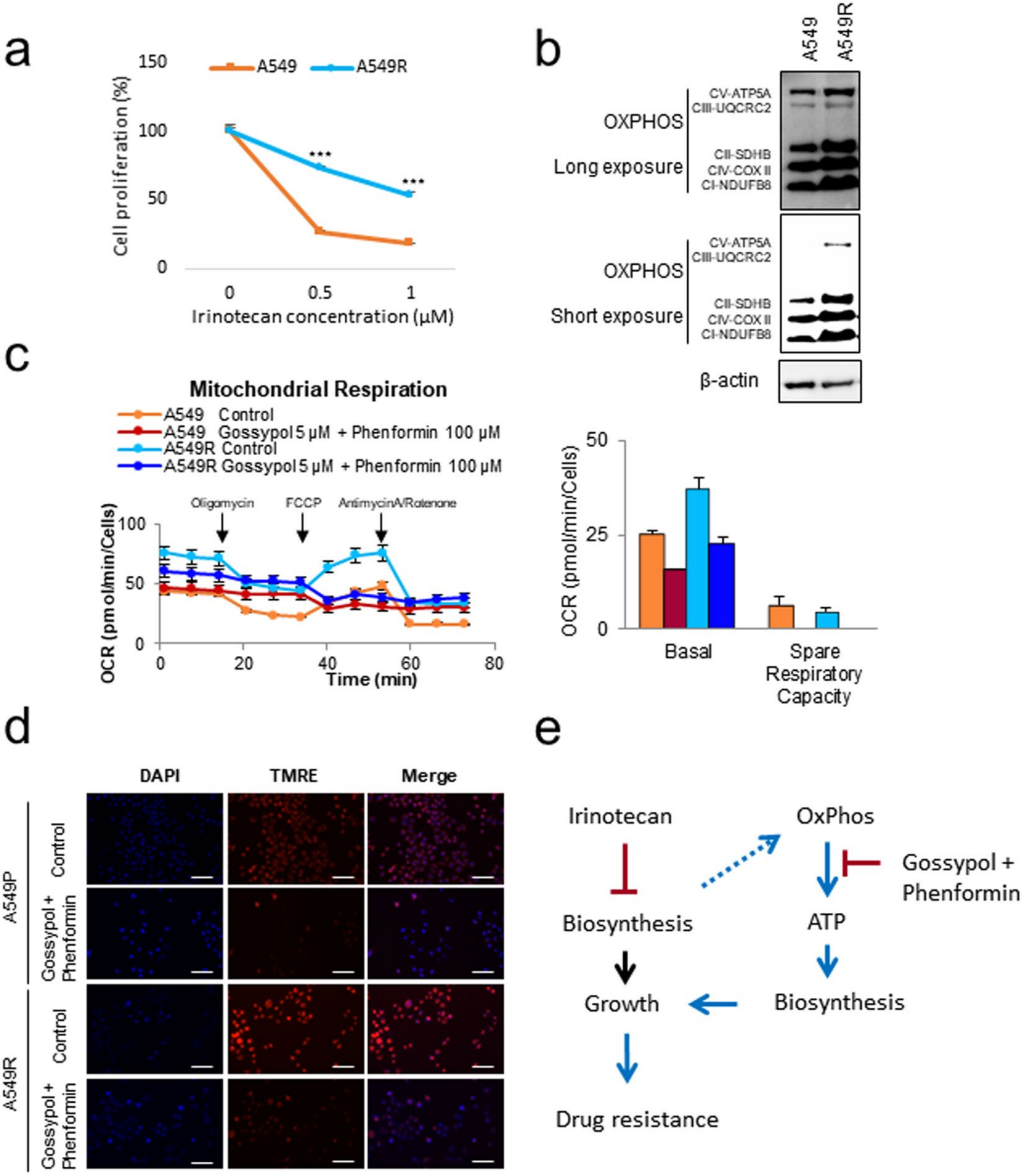
Cell Oxygen Consumption Rate in A549R cells is increased compared to A549 cells. (**a**) The effect of irinotecan on irinotecan-resistant A549 (A549R) cells after 48 h was determined by SRB assay (n = 3). (**b**) Levels of mitochondrial OxPhos complexes were increased in A549 irinotecan-resistant cells compared to the control as determined by western blot. (**c**) Oxygen Consumption Rates and respiration parameters were analyzed in A549 and A549R cells after treatment of 5 μM gossypol and 100 μM phenformin for 24 h (n = 5). (**d**) Mitochondrial membrane potential was analyzed by live cell imaging in A549 and A549R cells after treatment of 5 μM gossypol and 100 μM phenformin for 24 h. Scale bar = 100 µm. (**e**) Strategy for overcoming irinotecan resistance by OxPhos inhibition using gossypol and phenformin. The mechanism is not revealed how biosynthesis suppression by irinotecan induces OxPhos. Each bar represents the mean + s.d. *p < 0.05, **p < 0.01, ***p < 0.001.

We have selected the irinotecan resistant A549 cell line by serial increase of irinotecan treatment in the culture media for 10 passages. The irinotecan resistant A549 cell line (A549R) showed about 3-fold higher cell proliferation capacity compared to the sensitive A549 parental cell line at 0.5 µM irinotecan treatment (Fig. 1a). A549R cells showed no resistance to other anti-cancer drugs (Figure S2). Immunoblotting of OxPhos components using antibody cocktails showed increased levels of complexes I, II, III, IV, and V in A549R compared to the level of A549 (Fig. 1b). Mitochondrial respiration analysis revealed that the A549R cancer cell showed increase in the basal oxygen consumption rate (OCR) compared to A549 (Fig. 1c). It was confirmed that the components of OxPhos were also upregulated in A549R by immunoblotting compared to the A549 (Fig. 1b). Blocking OxPhos using gossypol and phenformin combination resulted in decrease of OCR as much as 40% (Fig. 1c). To test mitochondrial activity, the mitochondrial membrane potential was measured by tetramethylrhodamine ethyl ester (TMRE) staining (Fig. 1d). We observed highly activated mitochondrial membrane potential in A549R using TMRE staining compared to A549 (Fig. 1d). By treatment of gossypol and phenformin, mitochondrial OxPhos was significantly decreased in the A549R cell line (Fig. 1d). Therefore, we can summarize this observation as in Fig. 1e. Stress induced by biosynthesis suppression with irinotecan may increase OxPhos, which may increase biosynthesis through ATP synthesis as a recurrent growth of cancer (Fig. 1e). Therefore, Oxphos inhibition using gossypol and phenformin may block the salvage pathway of biosynthesis in cancer.

### OxPhos inhibition reversed irinotecan resistance as well as increased anti-cancer effect synergistically with irinotecan in NSCLC

**Figure 2:**
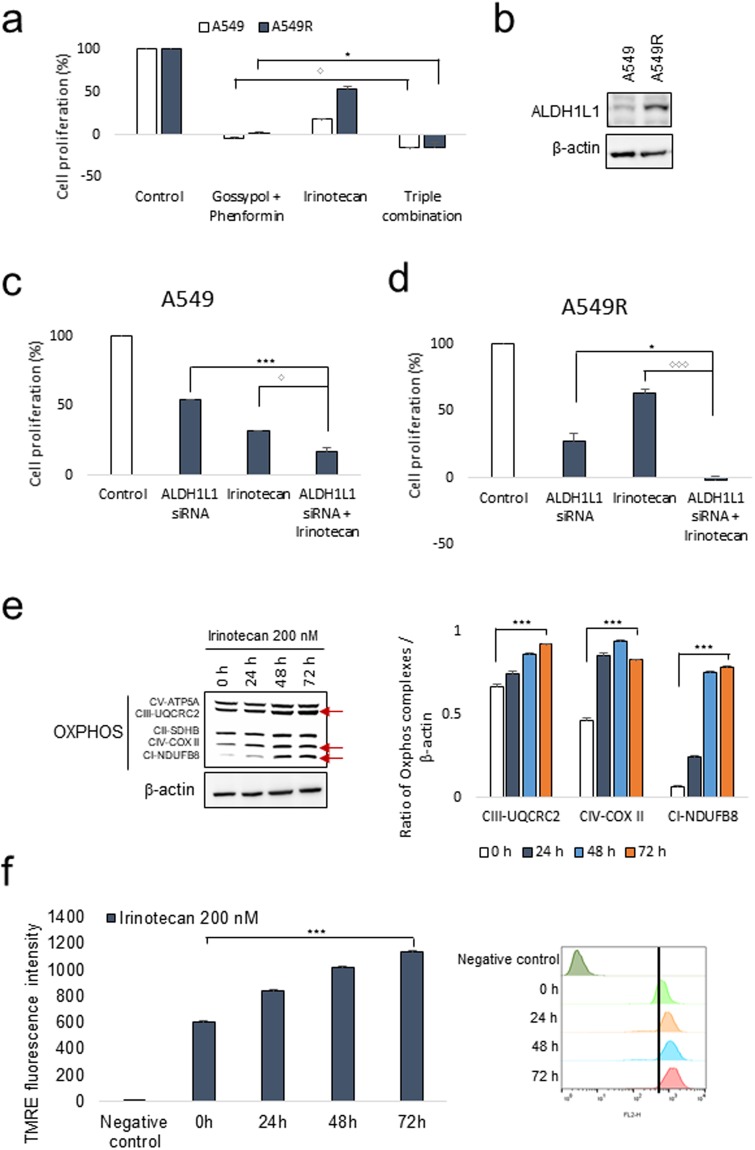
OxPhos inhibition sensitizes irinotecan-resistant cells to irinotecan. (**a**) The effect of 5 μM gossypol and 100 μM phenformin on irinotecan-resistant A549 (A549R) cells after 48 h was determined by SRB assay (n = 3). (**b**) Increased protein level of ALDH1L1 on irinotecan-resistant A549 (A549R) cells as analyzed by immunoblotting. (**c**–**d**) The effect of 40 μM ALDH1L1 siRNA treatment for 72 h and irinotecan treatment for 48 h on A549 and A549R cells was determined by SRB assay (n = 3). (**e**) Increased protein levels of oxphos complexes were analyzed after transient treatment of 200 μM irinotecan on A549 cells by immunoblotting. (**f**) Transient treatment of 200 μM irinotecan on A549 cells increased mitochondrial membrane potential, as determined by TMRE staining and flow cytometry (n = 3). Each bar represents the mean + s.d. *p < 0.05, **p < 0.01, ***p < 0.001.

NSCLC cancer cells adopted a special source of electron for ATP synthesis through OxPhos from cytosolic NADH produced by ALDH1L1 in the one carbon pathway (Figure S1)^7–9^. Gossypol inhibits ALDHs, and phenformin inhibits mitochondrial electron transfer complex I. Normal cells have not been affected by gossypol, because the cells use mitochondrial NADH produced from TCA cycle instead of ALDH. Normal cells also have not been significantly affected by phenformin, because they can use the electron complex II for electron entry, while cancer cells depend on complex I for ATP generation, redox homeostasis, and reactive oxygen species production^11^. Therefore, gossypol and phenformin did not affect ATP production in normal cells or the growth of normal tissue during treatment, while reducing ATP production down to 20% level of control^7,10^. To test whether gossypol and phenformin reduced irinotecan resistance, cell proliferation was measured by SRB test (Fig. 2a). A549R showed irinotecan resistance but it was sensitized by gossypol and phenformin to triple combination (Fig. 2a). The irinotecan resistance concurred to the level of ALDH1L1 in A549R (Fig. 2b). By ALDH1L1 knockdown using siRNA, cell proliferation was significantly reduced both in A549 and A549R (Fig. 2c,d). Combination treatment of irinotecan with ALDH1L1 knockdown showed a synergistic anti-proliferation effect in A549 as well as in irinotecan-resistant A549R (Fig. 2c,d). Increased level of mitotracker staining was observed in the irinotecan resistant cell A549R compared to the control wild type A549 (Figure S3).

OxPhos components and ALDH1L1 expressions were increased in A549R cells. To test whether the elevated OxPhos was acquired for establishing A549R cells, expression levels of OxPhos components were measured followed by transient irinotecan treatment (24~72 h) of normal A549 cells. The expressions of these proteins as well as mitochondrial membrane potential were analysed in A549 cells after transient treatment of irinotecan. The level of OxPhos complex I was increased up to 10-fold and mitochondrial membrane potential was increased up to 2-fold after 72 h treatment (Fig. 2e,f). These data suggested that these elevated expressions were acquired during establishing A549R cells.

**Figure 3:**
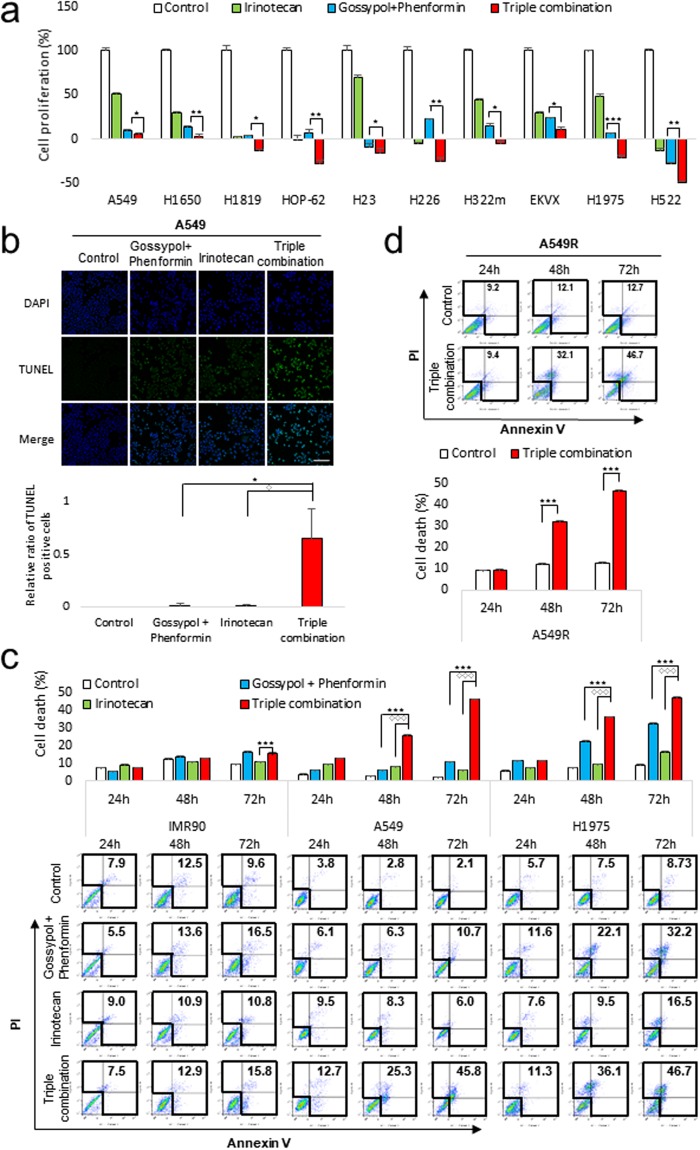
Triple-combined treatment of gossypol, phenformin with irinotecan shows a synergistic effect on cell viability reduction and cell death induction in NSCLC cells. (**a**) Synergistic effect of triple-combined treatment of 5 μM gossypol, 100 μM phenformin and 1 μM irinotecan after 48 h on cell proliferation was determined by SRB assay (n = 3). (**b**) Synergistic effect of triple-combined treatment after 24 h on cell death was determined by TUNEL assay (n = 4). Scale bar = 100 µm. (**c)** Synergistic effect of triple-combined treatment on cell death was determined by Flow cytometry analysis (n = 3). Each bar represents the mean + s.d. *p < 0.05, **p < 0.01, ***p < 0.001. (**d**) Effect of triple-combined treatment on cell death of A549R was determined by Flow cytometry analysis (n = 3). Each bar represents the mean + s.d. *p < 0.05, **p < 0.01, ***p < 0.001.

To test whether OxPhos inhibition using gossypol and phenformin generally sensitizes irinotecan effect on cell proliferation in NSCLC cell lines, 10 NSCLC cell lines were tested (Fig. 3). All cell lines showed near total growth arrest using triple combination, although A549, H23, H322m, and H1975 showed about 50% growth inhibition in treatment using only irinotecan (Fig. 3a). However, other anti-cancer drugs such as doxorubicin, cisplatin and 5-FU did not show synergistic effect with gossypol and phenformin (Figure S4a). Gemcitabine showed a significant synergistic effect with gossypol and phenformin *in vitro* cell culture assay but did not have synergistic effect *in vivo* xenograft model (Fig. S4b,c).

Growth arrest induced cell death concurred with the synergistic effect of triple combination by TUNEL staining (Fig. 3b). By FACS analysis, cell death was increased only by triple combination treatment in a time-dependent manner (Fig. 3c). Irinotecan with gossypol and phenformin treatment showed about a 4-fold increase of cell death after 72 h, while irinotecan increased cell death about 1~2-fold after 72 h treatment (Fig. 3c). A549R cells were also sensitive to triple combination treatment with a 4-fold increase in cell death (Fig. 3d). Triple combination showed no cell death induction in IMR-90 until 48 h (Fig. 3c).

### OxPhos inhibition with irinotecan treatment triggers a novel cell death pathway

**Figure 4:**
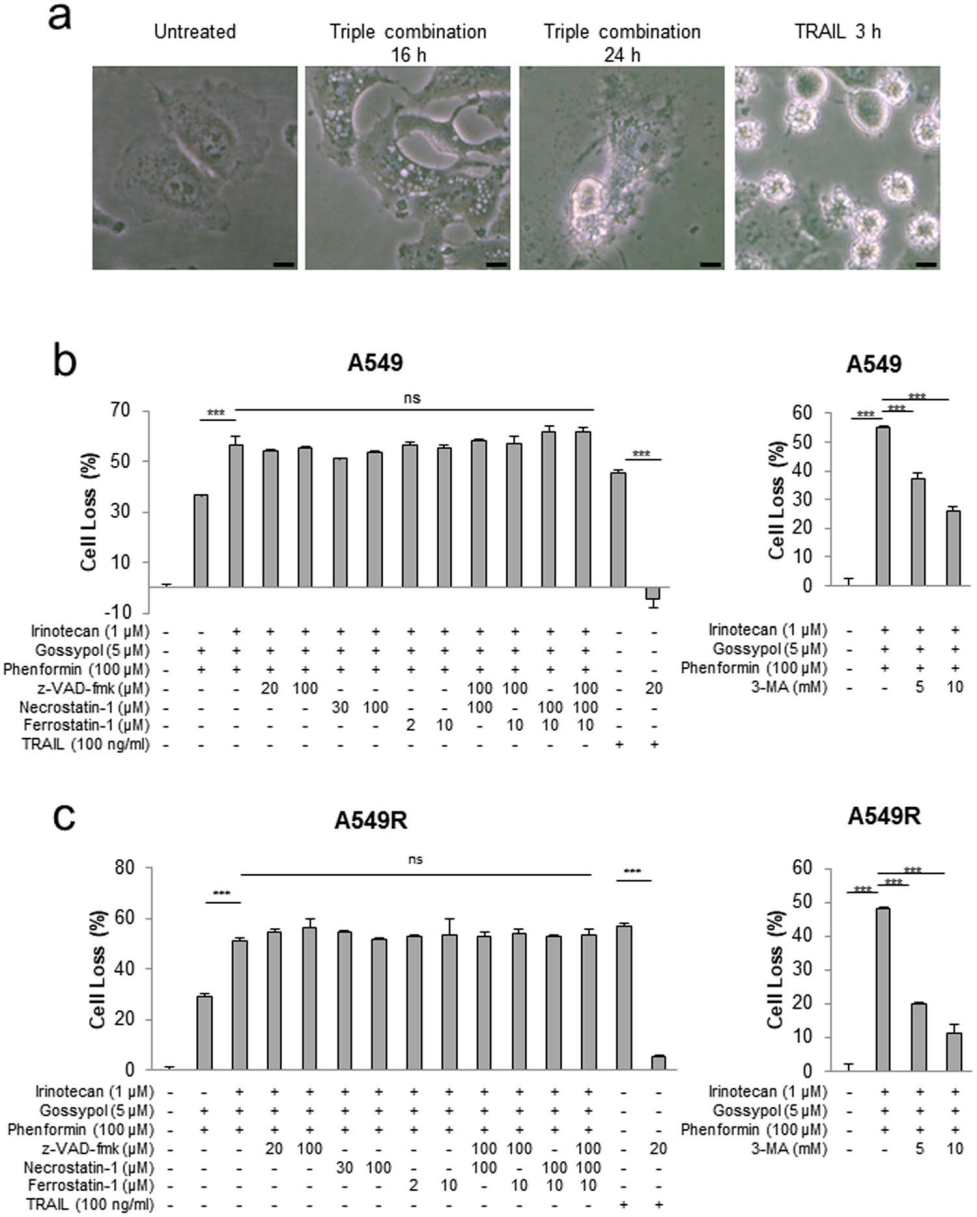
The cell death induced by triple combination treatment showed a morphology which is not a typical mode of cell death. (**a**) A549 cells were treated with triple-combination of 5 μM gossypol, 100 μM phenformin and 1 μM irinotecan, or 200 ng/ml TRAIL for indicated times, and then cells were imaged by means of a microscope. Scale bar = 10 µm. (**b**,**c**) A549 cells were treated with combination of irinotecan, gossypol, phenformin, z-VAD-fmk, necrostatin-1, ferrostatin-1 and 3-MA for 24 h. Cells were incubated with Cell Titer-Glo for 20 min, and then cell loss was analyzed by luminometer (n = 3). TRAIL is used as a positive control. Each bar represents the mean + s.d. NS no significance; ***P < 0.001.

To determine the mode of cell death induced by triple combination treatment, we compared the morphology of cell death induced by triple combination treatment with apoptotic cells. As shown in Fig. 4, apoptotic cells induced by TRAIL showed cell shrinkage and chromatin condensation during cell death. On the other hand, A549 cells treated with triple combination using irinotecan, gossypol, and phenformin showed the increase of granule and swelling of cells, and finally, this resulted in plasma membrane rupture (Fig. 4a). These features of cell death are more similar to necrosis or autophagic cell death than non-lytic cell death, apoptosis. Next, to know whether triple combination treatment induces other cell death mode rather than apoptosis, we treated several cell death inhibitors with triple combination treatment (Fig. 4b,c). As shown in Fig. 4b, the high dose of z-VAD-fmk (pan-caspase inhibitor), Necrostatin-1(RIPK1 inhibitor; Necroptosis inhibitor), Ferrostatin-1(Ferroptosis inhibitor) failed to inhibit triple combination induced cell death. To test whether autophagic cell death is involved in triple combination induced cell death, we treated 3-MA which is autophagy inhibitor (Fig. 4c). Interestingly, 3-MA partially inhibited triple combination induced cell death, suggesting that the cell death induced by triple combination treatment seems to be autophagic cell death rather than other cell death mode. Cell proliferation of irinotecan resistant A549R cell was about 3-fold higher compared to the proliferation of wild type A549 at 1 μM by SRB analysis (Fig. 1a). Triple combination treatment with gossypol (5 μM) and phenformin (100 μM) abolished the 3-fold resistance of A549R to irinotecan (1 μM) by Cell Titer-Glo Luminescent Assay (Fig. 4c). Triple combination treatment showed the same effect of cell death to A549R cells as shown in A549 cells (Fig. 4c) that is concurred with Fig. 2a measured by SRB analysis (Fig. 2a).

### Irinotecan treatment combined with gossypol and phenformin demonstrated a remarkable therapeutic response in NSCLC

**Figure 5:**
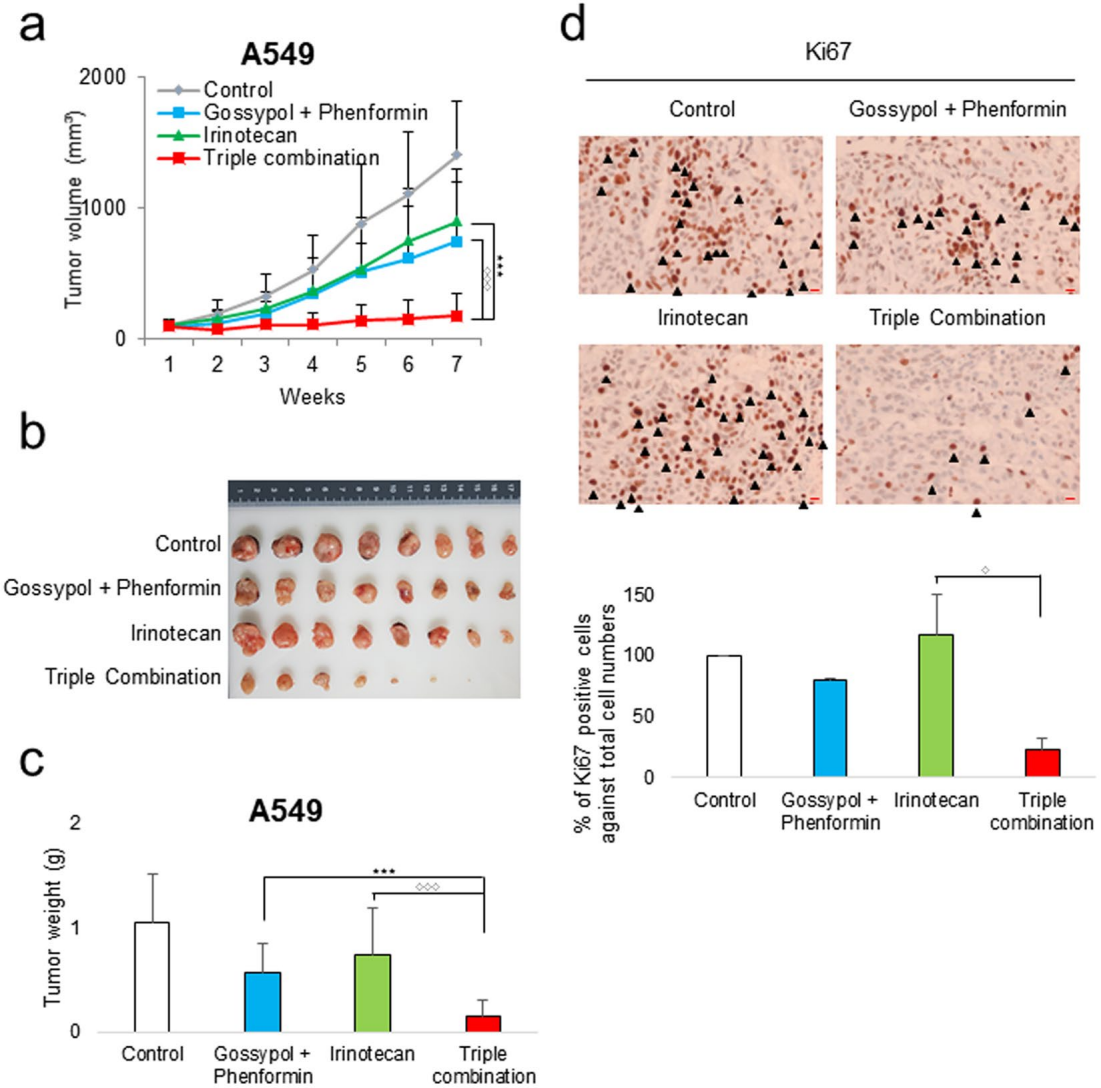
Growth inhibition of human NSCLC tumor xenografts by triple combination treatment of gossypol, phenformin and irinotecan. (**a**) A549 cells (7.5 × 10^6^) were injected in 6 weeks old BALB/c nude mice. When the volume of the tumor mass reached approximately 100 mm^3^, the mice were randomly assigned to one of the four treatment groups as indicated in the graph (n = 8). Gossypol (80 mg/kg) and phenformin (100 mg/kg) were administered orally once per day, 6 days/week and irinotecan (20 mg/kg) intraperitoneally 1 day/week. Graph shows a synergistic decrease in tumor growth after triple-combined treatment as measured using calipers. (**b**) Representative photograph of subcutaneous tumors derived from A549. (**c**) Final weight of subcutaneous tumors derived from A549. (**d**) IHC images of Ki67 staining of subcutaneous tumor sections derived from A549. Scale bar = 20 µm. Each bar represents the mean + s.d. *p < 0.05, **p < 0.01, ***p < 0.001.

**Figure 6:**
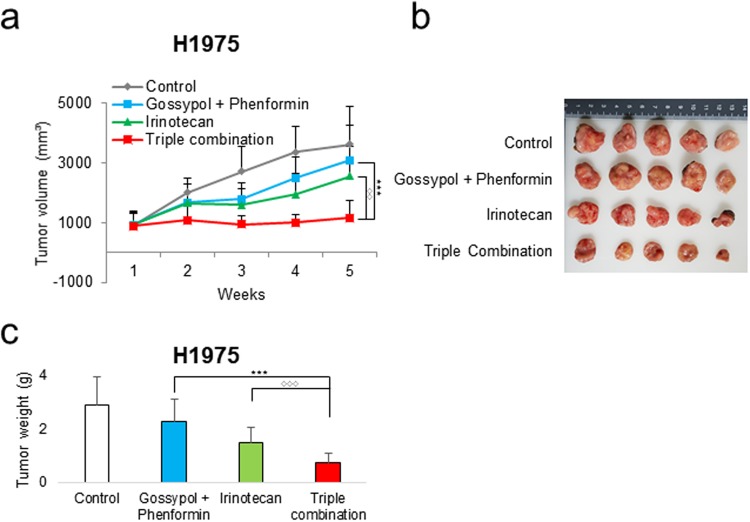
Growth inhibition of human NSCLC tumor xenografts by triple combination treatment of gossypol, phenformin and irinotecan. (**a**) H1975 cells (5 × 10^6^) were injected in 6 weeks old BALB/c nude mice. When the volume of the tumor mass reached approximately 1000 mm^3^, the mice were randomly assigned to one of the four treatment groups as indicated in the graph (n = 5). Gossypol (80 mg/kg) and phenformin (100 mg/kg) were administered orally once per day, 6 days/week and irinotecan (40 mg/kg) intraperitoneally 1 day/week. Graph shows a synergistic decrease in tumor growth after triple-combined treatment as measured using calipers. (**b**) Representative photograph of subcutaneous tumors derived from H1975. (**c**) Final weight of subcutaneous tumors derived from H1975. Each bar represents the mean + s.d. *p < 0.05, **p < 0.01, ***p < 0.001.

Irinotecan treatment combined with gossypol and phenformin for 72 h induced cell death by 4-fold in A549 and H1975 cells compared to the control while single treatment of irinotecan induced cell death up to 2-fold in H1975 cell compared to the control (Fig. 3c). We tested whether irinotecan treatment combined with gossypol and phenformin produced any synergistic therapeutic effect in the NSCLC mouse xenograft model (Figs 5 and 6). Cultured A549 cells were injected subcutaneously near the scapulae of 8-week-old female nude BALB/c mice. Oral administration of gossypol (80 mg/kg) and phenformin (100 mg/kg), and i.p. administration of irinotecan (20 mg/kg) was initiated when tumors reached a volume of 100 mm^3^ and was continued for 7 weeks. No physical toxicity was observed in mice that received the combination treatment for 2 weeks (Figure S5a). Single administration of irinotecan or combination treatment of gossypol and phenformin showed about 50% tumor growth arrest compared to the non-treated control (Fig. 5a–c). After 7 weeks of treatment, tumor volumes were reduced significantly with triple combination therapy compared to vehicle-treated control as well as single irinotecan-treated group, clearly demonstrating the enhanced therapeutic efficacy of triple combination *in vivo* (Fig. 5a–c). Tumor proliferation was measured by immunohistochemical staining of Ki67, which resulted in 5-fold decrease of Ki67 positive cells in the triple combination group while about 40% increase of Ki67 positive cells in the irinotecan group (Fig. 5d).

To test whether triple combination has a cytotoxic effect of tumor growth, triple combination treatment was started after tumor volume reached to 1000 mm^3^ using H1975 cell (Fig. 6). After 5 weeks, non-treated control reached to 3000 mm^3^ volume that had to be terminated due to IACUC guide line (Fig. 6a). Irinotecan (40 mg/kg) alone or combination of gossypol (80 mg/kg) and phenformin (100 mg/kg) was not enough to stop growing tumors (Fig. 6a–c). However, triple combination showed no growth of tumor from 1000 mm^3^ for 5 weeks without weight loss of mice (Figure S5b).

## Discussion

We found that irinotecan resistance is associated with increased mitochondrial OxPhos in NSCLC, which can be reversed by OxPhos inhibition using gossypol and phenformin. Irinotecan-based chemotherapy has already been shown to be effective against colorectal cancer, small cell lung cancer, and gastric cancer^12–14^. The overall survival rate of irinotecan-based chemotherapy is almost the same as other targeted therapeutic treatments^1^. However, a commonly confronted dilemma is the recurrence of cancer after targeted therapy limiting overall survival. Therefore, irinotecan treatment with gossypol and phenformin may have a therapeutic benefit to colorectal cancer and gastric cancer.

We have reported that combination of gossypol and phenformin induces cancer cell death following the decrease of ATP levels to 20% of control in NSCLC^7^. In this study, the synergistic therapeutic effect of triple combination using irinotecan has two important messages in NSCLC. One is that cell death induced by ATP depletion using gossypol and phenformin can be potentiated with irinotecan. We have employed two targets of ALDH1L1 and mitochondrial OxPhos complex I for a combinational inhibition to achieve cancer specific ATP depletion. ALDH1L1 is in one carbon pathway as a major electron supplier as NADH, which can be inhibited by gossypol^15^. OxPhos complex I is in the mitochondrial membrane as an electron carrier to ATP generator from NADH, which can be inhibited by phenformin or metformin^16,17^. The combinational treatment significantly reduced cancer mitochondrial oxidative phosphorylation, which can induce cell death^7^. Irinotecan does not induce cell death but induces cell cycle arrest^18^. Therefore, G2 cell cycle arrest by irinotecan may trigger synergistic damage to the cancer cell in the level of energy metabolism with gossypol and phenformin. Metabolite analysis after drug treatment may suggest a clue for the detailed mechanism in the near future. The second important message is that irinotecan resistance can be overcome by gossypol and phenformin treatment. There is a report that drug resistant neoplastic cells are critically dependent on oxidative phosphorylation rather than glycolysis in melanoma^19^. BRAF inhibitor leads to cell-cycle arrest and apoptosis, which triggers induction of the mitochondrial master regulator, PGC1α^19^. Later, cancer cells treated with BRAF inhibitor renders cancer cells addicted to the increased OxPhos^19^. DNA damaging agents including irinotecan also triggers induction of mitochondrial OxPhos^20^. The adaptive metabolic program limiting the efficacy of anti-cancer drugs is cancer energy metabolism. Therefore, a chance of recurrence from irinotecan treatment in NSCLC can be abrogated, by suppression of cancer mitochondrial oxidative phosphorylation through cancer ATP production, down to the 20% level of control using gossypol and phenformin^7^.

We observed that irinotecan treatment to the wild type cancer cells induces OxPhos components in a time dependent manner, which becomes irinotecan resistance cancer cells. We do not have clue whether OxPhos increase is ahead of irinotecan resistance or irinotecan resistance is ahead of OxPhos increase. However, they are tightly associated to acquire irinotecan resistance because OxPhos inhibition reverses irinotecan resistance. The mechanism of OxPhos increase by irinotecan treatment remains to be revealed to understand how cancer cell can evade drug toxicity by increase of OxPhos components. Human mitochondrial DNA (mtDNA) encodes a subset of the components for OxPhos^21^. These mRNAs are transcribed and then translated within the mitochondrial matrix by a unique machinery. Two mitochondrial ribosomal RNAs (the RNA components of the mitochondrial gene expression system) are also encoded by mtDNA, whereas all other protein components are encoded by nuclear genes and imported into mitochondria from the cytosol^21^. Report suggested that about 300 nucleus-encoded proteins are dedicated to serve mitochondrial gene expression^22,23^. This includes RNA polymerase and transcription factors, endonucleases for RNA precursor processing, aminoacyl-tRNA synthetases, RNA-modifying enzymes and biogenesis factors for the mitochondrial ribosome and translation factors^21^. This suggests that increase of OxPhos components by irinotecan treatment may be associated with multiple transcription and translation regulations.

Another important finding in this study is that irinotecan enhanced cancer cell death when combined with gossypol and phenformin treatment, even though the cell cycle arrest itself by irinotecan does not induce much of cell death *in vitro*^18^. In this study, we have blocked several death pathways using z-VAD-fmk for apoptosis, NCC-1 for necroptosis, NAC for ferroptosis while NSCLC was treated with triple combination. None of them could block cancer cell death induced by gossypol and phenformin. However, 3-MA partially inhibited triple combination induced cell death, suggesting that the cell death induced by triple combination treatment likely depends on autophagic cell death rather than other cell death mode.

## Electronic supplementary material

Supplementary Figures
