# Supplementary material for: Targeting Mitochondrial Oxidative Phosphorylation Abrogated Irinotecan Resistance in NSCLC
Source: Sci Rep. 2018 Oct 24;8:15707. doi: 10.1038/s41598-018-33667-6 (PMC6200737; doi:10.1038/s41598-018-33667-6)
Supplement: Supplementary file 1 — Supplementary Figures [file 41598_2018_33667_MOESM1_ESM.pdf]

Supplementary information

Targeting Mitochondrial Oxidative Phosphorylation Abrogated Irinotecan Resistance in NSCLC

Soohyun Lee[1,2], Jae-Seon Lee[1], Jinho Seo[2], Seon-Hyeong Lee[1], Joon Hee Kang[1], Jaewhan Song[2,3] and Soo-Youl Kim[1,4]

[1]Tumor Microenvironment Research Branch, Division of Cancer Biology, Research Institute, National Cancer Center, Goyang 10408, Republic of Korea

[2]Department of Biochemistry, College of Life Science and Biotechnology, Yonsei University, Seoul 03722, Korea.

[3]Co-corresponding author: Tel: +82-2-2123-5695; Fax: +82-362-9897; E-mail: jso678@yonsei.ac.kr

[4]Corresponding author: Tel: +82-31-920-2229; Fax: +82-31-920-2006; E-mail: kimsooyoul@gmail.com

Figure S1

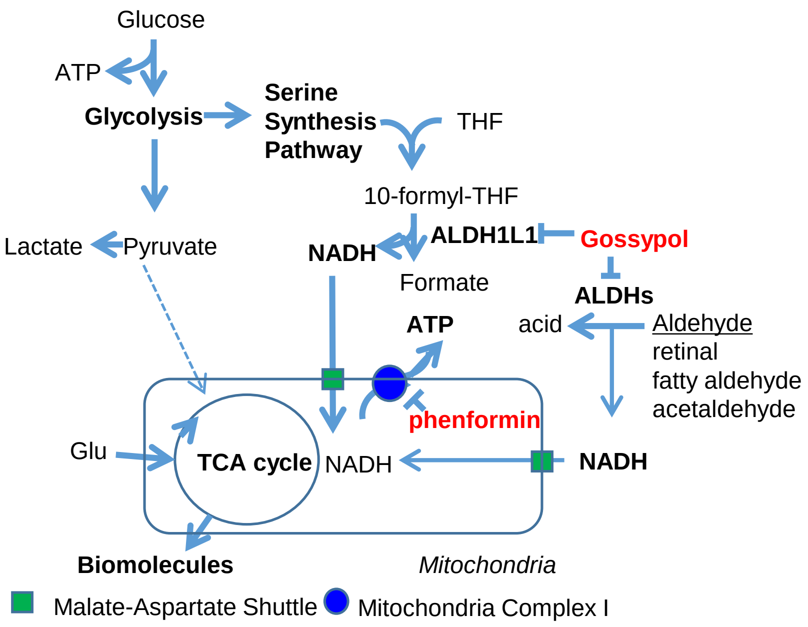

Figure S1. Proposed model for the role of ALDH1L1 in ATP synthesis through NADH production.

Figure S2

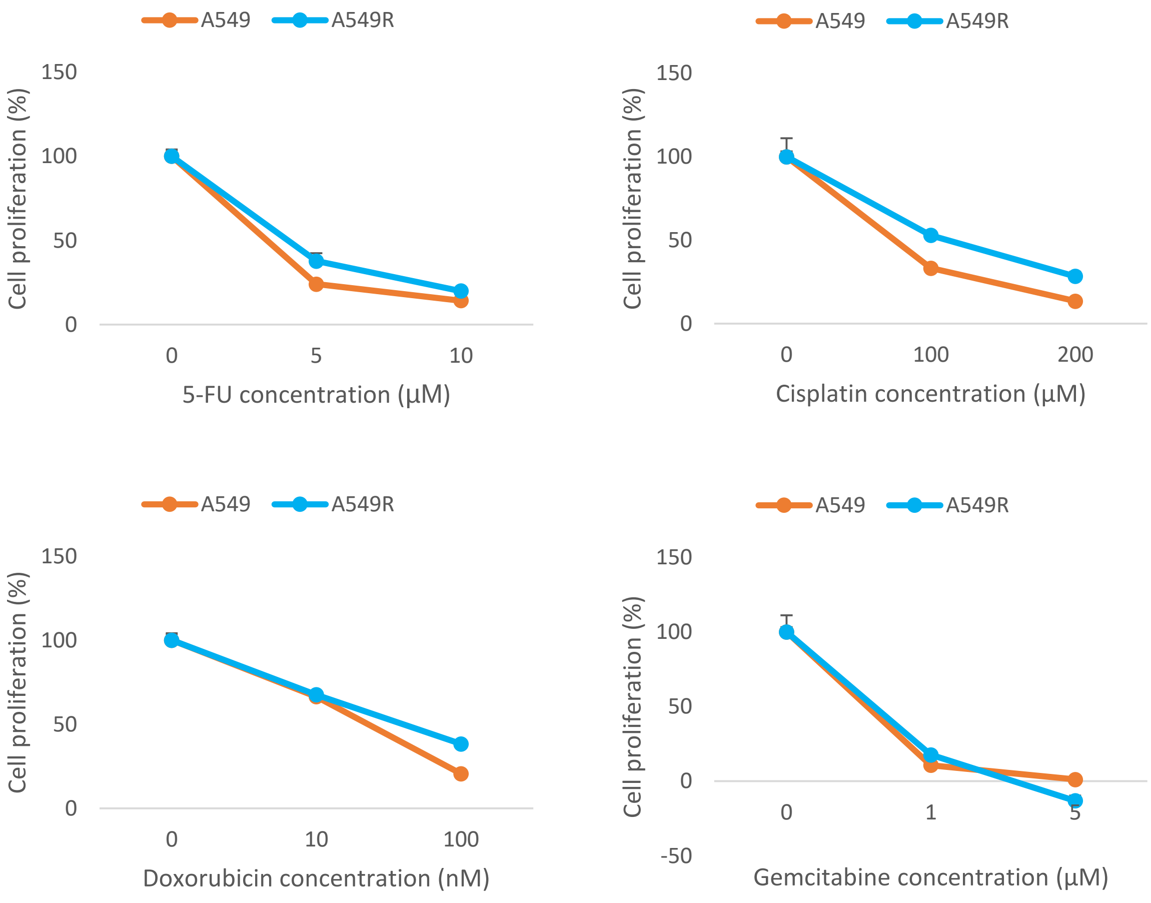

Figure S2. A549R cells are resistant to irinotecan and no other anticancer drugs.

A549 and A549R cells were treated with different anticancer drugs for 48h as indicated and the effect on cell proliferation was determined by SRB assay.

Figure S3

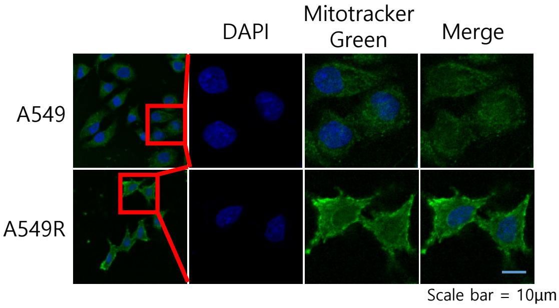

Figure S3. Images of A549 and A549R cells after mitotracker green staining. Scale bar = 10 μm.

Figure S4

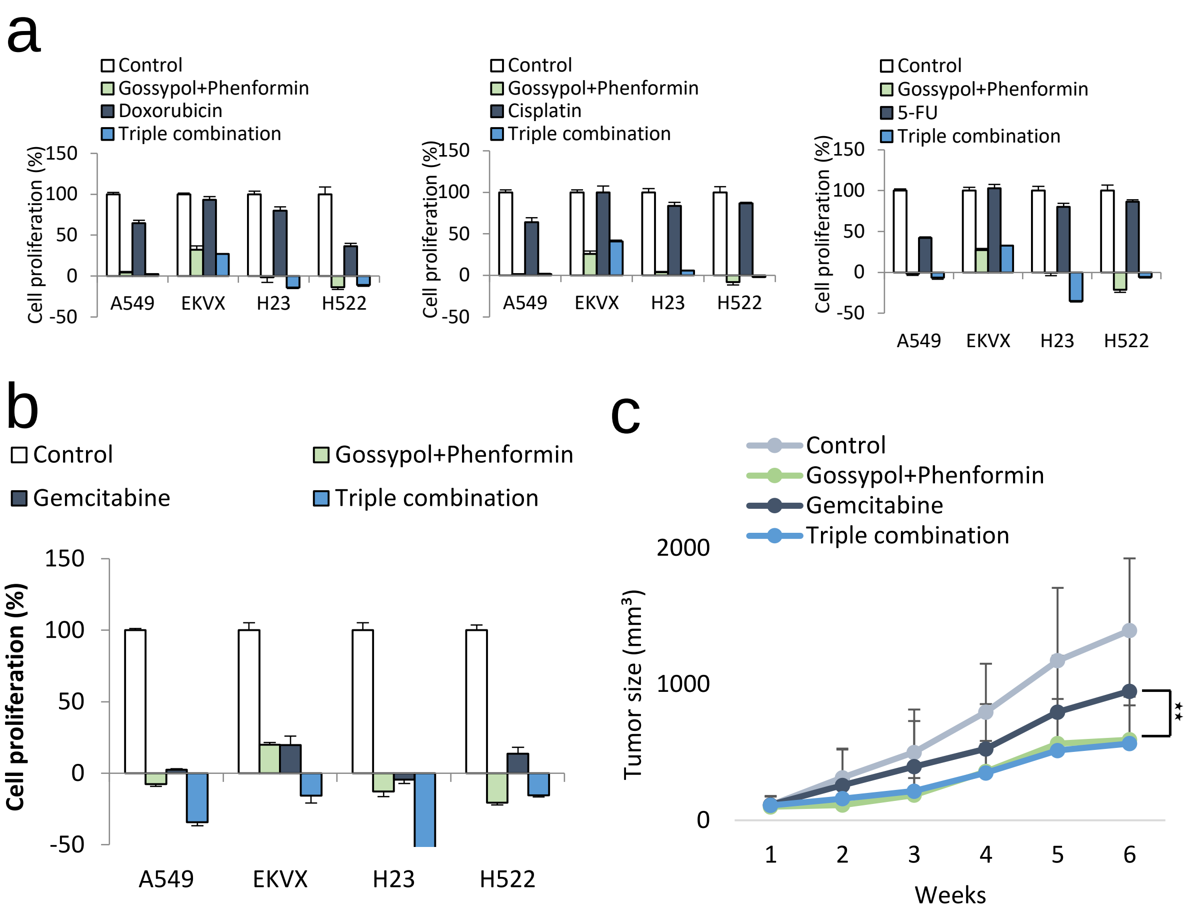

Figure S4. Triple combination treatment of gossypol and phenformin with gemcitabine showed no synergistic effect on xenograft mouse model. a Triple-combined treatment of 5 μM gossypol, 100 μM phenformin with different anticancer drugs (200 μM cisplatin, 5 μM gemcitabine and 100 nM doxorubicin) after 48 h showing no synergistic effect on cell proliferation as determined by SRB assay. **b** Synergistic effect of triple-combined treatment of 5 μM gossypol, 100 μM phenformin and 1 μM gemcitabine after 48 h on cell proliferation was determined by SRB assay. **c** A549 cells (7.5 X 10^6) were injected in 6 weeks old BALB/c nude mice. When the volume of the tumor mass reached approximately 100 mm^3, the mice were randomly assigned to one of the four treatment groups as indicated in the graph (n=8). Gossypol (80mg/kg) and phenformin (100mg/kg) were administered orally once per day, 6 days/week and gemcitabine (80 mg/kg) intraperitoneally 1day/week. Graph shows a synergistic decrease in tumor growth after triple-combined treatment as measured using calipers. Each bar represents the mean + s.d.

Figure S5

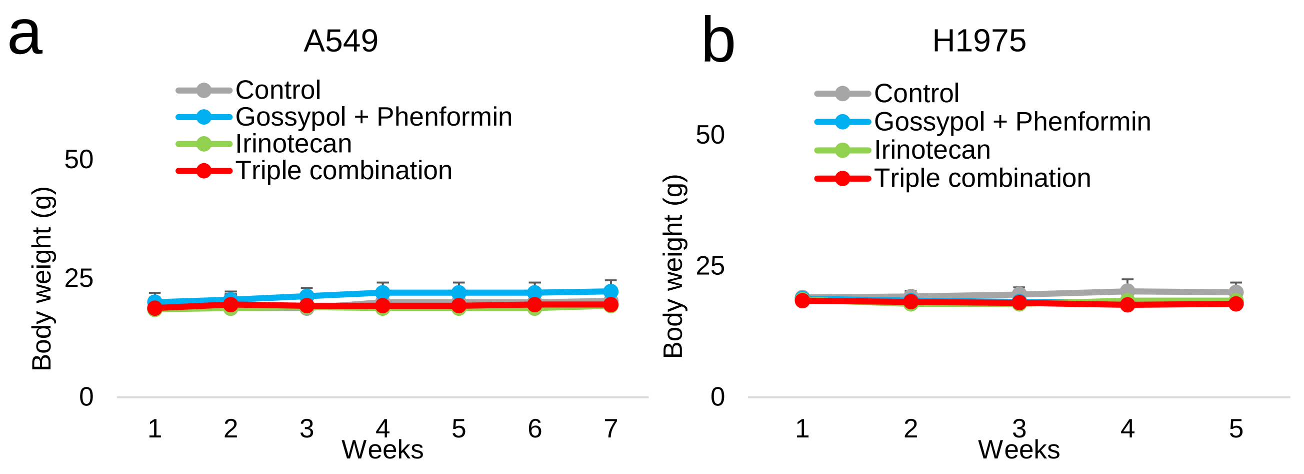

Figure S5. No physical toxicity was observed in mice after triple-combination treatment in xenograft mouse model.
a Body weight of mice of four treatment groups after A549 inoculation were measured every week (n=8). **b** Body weight of mice of four treatment groups after H1975 inoculation were measured every week (n=5). Each bar represents the mean + s.d.

Full length gels for Figure 1b

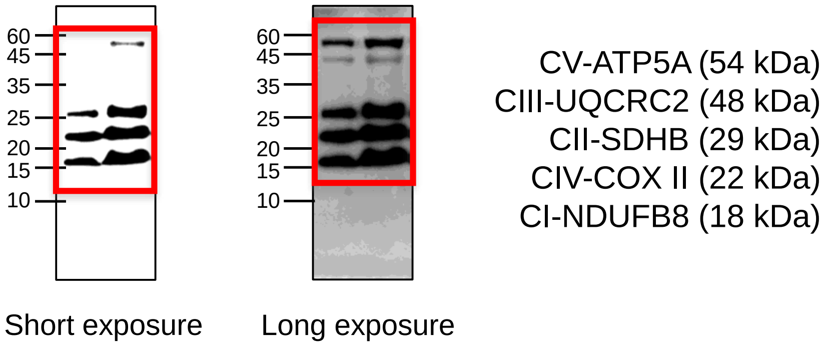

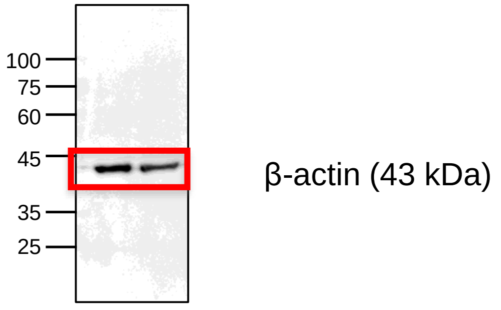

Areas of the gel shown in Figure 1b are marked in red. Predicted molecular weights for OXPHOS complexes and β-actin are as indicated.

Full length gels for Figure 2b

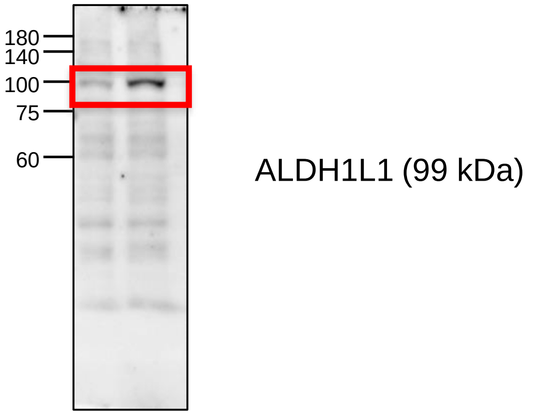

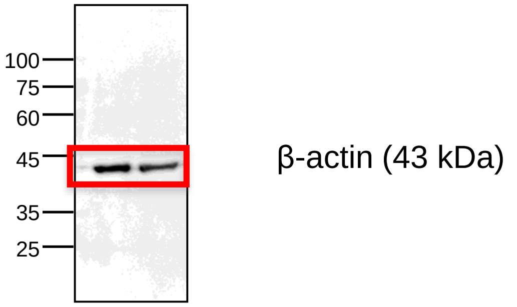

Areas of the gel shown in Figure 1b are marked in red. Predicted molecular weights for ALDH1L1 and β-actin are as indicated.

Full length gels for Figure 2e

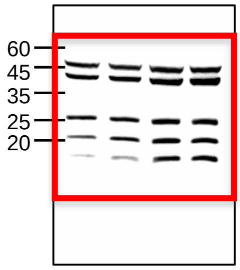

CV-ATP5A (54 kDa)
CIII-UQCRC2 (48 kDa)
CII-SDHB (29 kDa)
CIV-COX II (22 kDa)
CI-NDUFB8 (18 kDa)

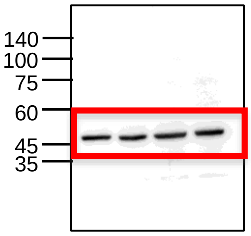

β-actin (43 kDa)

Areas of the gel shown in Figure 2e are marked in red. Predicted molecular weights for OXPHOS complexes and β-actin are as indicated.
